# Tantalizing Glimpses into a Fragmented Genome

**DOI:** 10.1371/journal.pbio.1001470

**Published:** 2013-01-29

**Authors:** Mary Hoff

**Affiliations:** Freelance Science Writer, Stillwater, Minnesota, United States of America

*Oxytricha trifallax* is an unusual enough organism on the outside. Neither plant nor animal, covered with tiny hair-like protuberances, this one-celled protozoan cruises ponds and puddles in search of microbial meals. But even more unusual is what's inside.

**Figure:**
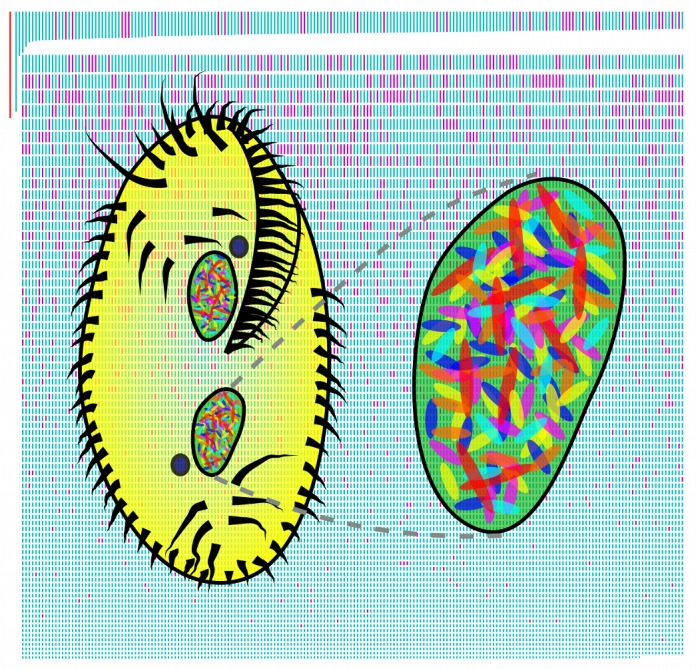
***Oxytricha*** **'s two macronuclei (green) and micronuclei (blue) are shown schematically over a size-ranked map of all nanochromosomes (alternatively fragmented chromosomes are fuchsia; the 70 kb mitochondrial genome is shown in red for scale).**

Like other ciliates, *O. trifallax* has two nuclei: a micronucleus, which contains its entire genome, and a macronucleus, which houses an edited version of the genetic material that's used to run the ship. What's extraordinary about *O. trifallax* is the degree to which its macronuclear genetic material has been rearranged. Derived afresh each generation from the micronuclear genetic material through an elaborate process that involves chopping up chromosomes, rearranging genes, deleting virtually all of the noncoding DNA, making multiple copies of the various bits, and capping them with telomeres, it ends up with tens of thousands of “nanochromosomes” that then serve as the templates for producing all the proteins that *O. trifallax* needs to conduct the day-to-day business of being alive.

*O. trifallax* and other ciliates have long been model organisms for studying cellular processes that take place in eukaryotes, including seminal studies in the 1980s on the structure of telomeres. The unusual processes *O. trifallax* goes through to create its macronucleus, and the complex result of these processes, creates a treasure-trove of potentially useful information for those studying the structure, manipulation, and function of genetic material in other organisms. As described in this issue of *PLOS Biology*, Estienne Swart, Laura Landweber, and colleagues successfully took up the challenge of assembling and analyzing the sequence of the incredibly complex *O. trifallax* macronuclear genome.

The researchers' efforts were rewarded with a number of surprising and enlightening discoveries. They found first of all that the *O. trifallax* macronucleus contains approximately 15,600 nanochromosomes and some 18,500 genes; as a consequence, 80 percent of the chromosomes contain only one gene each. As an extra source of complexity, about 10 percent of the nanochromosomes were alternatively fragmented, meaning that the different copies of each chromosome might contain different segments of genetic material. Nanochromosomes ranged in length from 469 to 66,000 base pairs, averaging about 3,200 base pairs. Each chromosome (and therefore gene) was present in approximately 2,000 copies, with the astounding consequence that each macronucleus is estimated to contain tens of millions of telomeres.

Curious about the relationship between nanochromosome structure and function, the researchers looked for potential correlations between fragment size and expression. They found that the DNA in shorter alternative nanochromosomes is more highly amplified than DNA from nanochromosomes that lack alternative fragmentation. However, copy number was not a strong predictor of expression level.

Exploring the difference in genetic material between the macronucleus and the micronucleus from which it was derived, the team learned that some 96 percent of the micronuclear DNA is discarded in the process of macronucleus formation. Nevertheless, various tests indicated that the macronuclear genome contains all of the genes needed for day-to-day cellular maintenance, with the exception of the transposon genes needed for sexual reproduction. Diversity in the macronuclear genome was found to be due not only to nucleotide polymorphisms carried over from the micronucleus, but also from additional changes taking place as the macronucleus formed. An assessment of allelic diversity indicated a very large effective population size, on the order of 26 million.

In search of clues to the mechanism behind the development of *O. trifallax's* unusual genome, the researchers discovered two new classes of transposase-like proteins unknown in other ciliates. If it turns out (as the researchers suspect) that these proteins are implicated in the reorganization of genes during macronuclear formation, *O. trifallax* could prove a valuable model for studying similar rearrangement processes that take place during evolution and in certain disease states. Similarly, the large number of telomeres present in the macronucleus continues to make it a valuable tool for studying these structures' origin and function.

The information gained during the arduous process of assembling and analyzing *O. trifallax's* highly fragmented, rearranged, and amplified genome provides useful insights into the biology of this unusual organism. More broadly, it provides, in the researchers' own words, “tantalizing glimpses into novel molecular biology and evolution.”

The research team is now following through by sequencing the *O. trifallax* micronuclear genome, which will presumably hold clues to the establishment of the telomeres and clarify the topological transformation effected during construction of the macronucleus. Together, the two genomes are expected to provide a valuable boost to our understanding of programmed gene rearrangement and other processes that not only give rise to *O. trifallax*'s unusual macronuclear architecture, but may also play key roles in shaping the structure and function of reorganized genes in other eukaryotes, including humans and organisms that harm or help us.

**Swart EC, Bracht JR, Magrini V, Minx P, Chen X, et al. (2013)** **The** ***Oxytricha trifallax*** **Macronuclear Genome: A Complex Eukaryotic Genome with 16,000 Tiny Chromosomes. doi:10.1371/journal.pbio.1001473**

